# Effect of Vest Load Carriage on Cardiometabolic Responses with Load Position, Load Mass, and Walking Conditions for Young Adults

**DOI:** 10.3390/bioengineering12020202

**Published:** 2025-02-18

**Authors:** Zhibo Jing, Hong Han, Jianda Han, Juanjuan Zhang

**Affiliations:** 1 Tianjin Key Laboratory of Intelligent Robotics, Institute of Robotics and Automatic Information System, Nankai University, Tianjin 300350, China; jzb@mail.nankai.edu.cn (Z.J.);; 2 College of Artificial Intelligence, Nankai University, Tianjin 300350, China; 3 School of Materials Science and Engineering, Smart Sensing Interdisciplinary Science Center, Nankai University, Tianjin 300350, China

**Keywords:** vest load carriage, cardiometabolic responses, load position, load mass, walking conditions

## Abstract

Carrying external loads, such as vest-borne systems, is common in occupations like firefighting and military service, yet the physiological impacts of load placement, mass, and walking conditions remain not fully understood. This study examined the effects of vest load carriage on metabolic rate and heart rate. Participants underwent three trials with varying load placements, masses (0–30 kg), and walking conditions (different speeds and inclines). Results showed no significant effect of load placement on metabolic and heart rates with a 10 kg vest load. When walking with a vest load at a speed of 5 km/h, the metabolic rate followed a quadratic relationship with load mass, while heart rate increased linearly. When walking with a 10 kg vest load, with slope ranging from 0% to 10% and speed ranging from 3 km/h to 7 km/h, each 5% increase in slope or 2 km/h increase in speed significantly elevated the metabolic rate. These findings highlight the importance of load mass in determining energy expenditure and can guide safer load-bearing designs.

## 1. Introduction

Carrying heavy equipment is a routine requirement in professions such as firefighting, law enforcement, and military service. These individuals often rely on load-bearing systems like backpacks, vests, and straps to manage their gear. While these systems are designed to distribute weight more effectively, external load carriage can still have significant physiological impacts on the body. Understanding how different load placements affect biomechanics and cardiopulmonary metabolism is crucial for improving the design of these systems, thereby enhancing performance. Similarly, in everyday life, many people, such as students and workers, also carry heavy loads as part of their daily activities. Furthermore, with the increasing use of wearable devices like exoskeletons that impose additional loads, it is becoming even more urgent to understand how such loads affect individuals, particularly those who are untrained.

Previous physiological studies have shown that external loading significantly alters gait biomechanics [1,2,3,4,5] and cardiorespiratory metabolism [6,7,8,9]. Even when the total load remains the same, changes in the load’s position can lead to significant differences in energy cost and heart rate [10,11,12]. For instance, loads placed closer to the body’s center of mass result in lower metabolic costs, while loads positioned on distal limbs greatly increase metabolic cost [13]. Specifically, each additional kilogram of weight placed on the feet increases metabolic cost by 7% to 10% [14], which is nearly double the metabolic cost of carrying the same weight on the thighs [15]. In contrast, carrying loads in the hands while walking does not significantly raise metabolic cost [16,17], and loads carried on the head result in the lowest energy cost [18,19].

Trunk load systems, such as backpacks, double packs, and vests, are common forms of load carriage. Extensive research has been conducted on the effects of backpack and double-pack loads on human physiology [20,21,22,23]. Backpack loads have been found to incur lower energy costs compared to other distal load configurations, and may even exhibit energy-saving phenomena under certain conditions [24]. The relationship between metabolic consumption and backpack load weight is approximately linear [8], and the position of the load’s center of mass within the backpack significantly affects metabolic cost [25]. Compared to backpack loads, double-pack systems tend to cause less gait disruption and result in smaller increases in metabolic consumption [11,23,26].

Load-bearing vests, similar to double-pack systems, distribute weight more evenly across the body and position the load’s center of mass closer to the trunk. This design reduces the impact on balance and gait. However, despite their widespread use, most research on vests has focused on the effects of load weight on cardiopulmonary metabolism [27,28], biomechanics [29], and physiology [30]. There has been limited investigation into how the position of the load within the vest affects energy expenditure, and little is known about the relationship between vest load weight and metabolic cost. Furthermore, the effects of different gait conditions under vest load-bearing on cardiopulmonary function are not fully understood.

The aim of this study was to comprehensively assess the effects of vest load placement, load mass, and walking conditions under load on the cardiopulmonary metabolism of young males. We applied a series of weighted loads via a vest at different locations on the trunk of young male participants. Subjects walked at various speeds and inclines while metabolic and heart rate measures were taken. By comprehensively assessing these factors, researchers can gain a more holistic understanding of the effects of vest loading on the cardiopulmonary function of young males, which holds significant implications for designing more effective load carriage systems or tasks within occupational settings.

## 2. Methods

### 2.1. Experimental Design

We conducted three separate trials to investigate the effects of load placement, load mass, and different walking conditions on cardiopulmonary metabolism while wearing a weighted vest.

#### 2.1.1. Load Placement Trial

**Figure 1:**
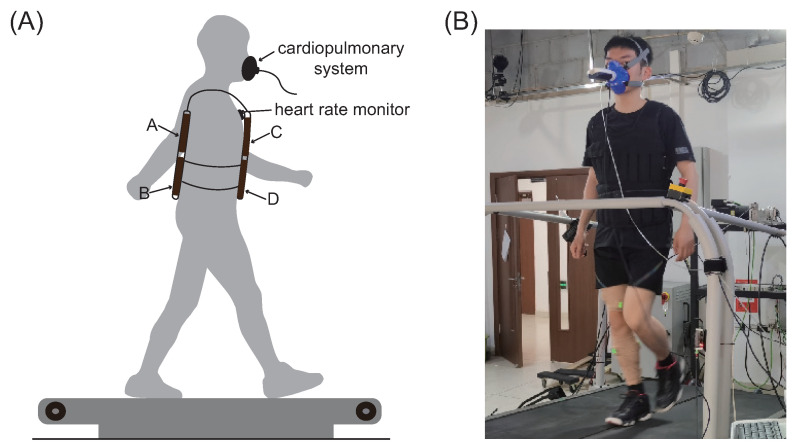
(**A**) In the load placement trial, six different load positions were tested: upper back (pocket A), lower back (pocket B), entire back (pocket A and B), upper torso (pocket A and C), lower torso (pocket B and D), and entire vest (pocket A, B, C, and D). (**B**) A participant is walking on the treadmill with a vest load.

In the load placement trial, six different load positions were tested (Figure 1): upper back (UB), lower back (LB), entire back (EB), upper torso (UT), lower torso (LT), and entire vest (EV). The load was varied by placing standard steel blocks (each weighing 0.25 kg) in the vest pockets to achieve the desired weight distribution. Participants walked on a treadmill (FIT, Bertec Corporation, OH, United States) that allowed for adjustable speed and incline settings to simulate various walking scenarios. The load mass was kept constant at 10 kg, the treadmill incline was set to 0%, and the walking speed was 5 km/h.

#### 2.1.2. Load Mass Trial

In the load mass trial, the load was uniformly distributed across the vest, with masses of 0 kg, 5 kg, 10 kg, 15 kg, 25 kg, and 30 kg. The treadmill incline was set to 0%, and the speed was 5 km/h. This walking speed was chosen based on [24], indicating minimal metabolic cost of the load carriage at approximately 5 km/h.

#### 2.1.3. Walking Conditions Trial

The walking conditions trial included variations in incline and speed. Participants walked at a constant speed of 5 km/h, while the incline increased from 0% to 5% and then to 10%. Additionally, participants walked at a constant incline of 0% while the speed increased from 3 km/h to 5 km/h and then to 7 km/h. The load was uniformly distributed across the vest (EV), with a mass of 10 kg.

### 2.2. Participants

We recruited thirty-one healthy young male adults (age 20.5 ± 1.8 years; height 176.3 ± 7.2 cm; body mass 68.5 ± 9.1 kg) to participate in this study. The participants were all in good physical condition and free of any cardiovascular or metabolic disorders that could affect the study outcomes. Data collection took place at the Human-Robot Interaction Gait Laboratory at Nankai University in China. All participants were Nankai University students. None had prior experience with weighted vests. Ten participants were assigned to the load placement trial, eleven to the load mass trial, and ten to the walking conditions trial. The study was approved by the Ethics Committee of Nankai University, and all participants provided written informed consent after being informed of the study’s purpose and potential risks.

### 2.3. Experimental Protocol

Participants attended a familiarization session 48 h before testing, practicing walking with a 5 kg load for 10 min to adapt to the vest. Research visits were conducted between 4:00 PM and 7:00 PM on each testing day. Participants were instructed to fast for more than 5 h prior to each visit and to avoid alcohol consumption for more than 24 h, intense exercise for more than 48 h, and both caffeine and nicotine for more than 24 h before testing. Fasting was required to ensure that participants were in a similar physiological state during each visit and to eliminate the thermic effect of food on metabolic measurements [31]. During testing, participants wore standard athletic training attire, including sports shorts, a T-shirt, socks, and the same model of athletic shoes.

Participants were asked to stand still for 6 min to measure their resting heart rate (HR) and metabolic rate ($E_{s}$) before starting each trial. Heart rate was continuously monitored using a wireless heart rate monitor (H10, Polar, Oulu, Finland) attached to the chest. A cardiopulmonary exercise system (HigherMed, Nanjing Gaomei Medical Technology Co., Ltd., Nanjing, China) was used to measure breath-by-breath oxygen uptake ($\dot{V}O_{2}$) and carbon dioxide exhalation ($\dot{V}CO_{2}$). Instantaneous metabolic rate was estimated by using a popular equation [32]:

(1) $$E_{i}=0.278\cdot \dot{V}O_{2}+0.075\cdot \dot{V}CO_{2}$$

where $E_{i}$ is the instantaneous metabolic rate in Watts, and $\dot{V}O_{2}$ and $\dot{V}CO_{2}$ are in mL/min.

Participants then proceeded with the walking tests, adhering to the specific conditions in each trial. Each walking condition was maintained for 6 min, with a 2 min rest period between conditions to minimize fatigue and allow participants to recover. The walking tests in each trial were repeated in reverse order, and the results from the two sequences were averaged to mitigate the effects of sensor measurement noise and metabolic or heart rate drift [33,34]. The metabolic rate for each condition was estimated based on the average values obtained during the final 3 min of each 6-minute walking interval. This approach was chosen to ensure that the data reflected steady-state conditions, minimizing the influence of initial transient responses. The net metabolic rate was defined as the difference between the metabolic rate during walking and the resting metabolic cost. To account for individual differences in body mass, the net metabolic rate was normalized by body weight:

(2) $$E_{net}=\frac{E_{w}-E_{s}}{M}$$

where $E_{net}$ is the net metabolic rate in W/kg, $E_{w}$ is the metabolic rate during walking in Watts, $E_{s}$ is the resting metabolic rate in Watts, and *M* is the participant’s body weight in kg.

### 2.4. Data Analysis

We used Matlab 2019b to perform data analysis. The mean, standard deviation, and coefficient of variation for metabolic rate and heart rate for each load position, load mass, and walking condition were calculated. One-way analysis of variance (ANOVA) was conducted on metabolic rate and heart rate across conditions in each trial. Post hoc pairwise comparisons with Bonferroni correction were performed.

For regression analysis, we employed linear and quadratic models to explore the relationships between mean metabolic cost and vest load mass, as well as between mean heart rate and vest load mass. The linear model assumed a direct, proportional relationship, while the quadratic model accounted for potential non-linear effects.

To determine the better fit between the models, we evaluated the goodness of fit ($R^{2}$) and the Akaike Information Criterion (AIC):

(3) $$\begin{array}{c} R^{2}=1-\frac{\sum_{i=1}^{n}{\left(y_{i}-{\hat{y}}_{i}\right)}^{2}}{\sum_{i=1}^{n}{\left(y_{i}-\bar{y}\right)}^{2}} \end{array}$$

where $y_{i}$ is a measured physiological metric (metabolic rate or heart rate) for the *i*-th load mass, ${\hat{y}}_{i}$ is the evaluated physiological value from a model (the linear model or the quadratic model) for the *i*-th load mass, $\bar{y}$ is the mean measured physiological metric across load mass, and n=7 is the number of load mass conditions. The $R^{2}$ value ranges from 0 to 1, with values closer to 1 indicating a better fit of the model to the data:

(4) $$\begin{array}{c} \mathrm{AIC}=2k-2\ln(L) \end{array}$$

where *k* is the number of estimated parameters in the model. *L* is the maximum likelihood estimate,

(5) $$\begin{array}{c} \ln L=-\frac{n}{2}\ln\left(2\pi \sigma^{2}\right)-\frac{1}{2\sigma^{2}}\sum_{i=1}^{n}{\left(y_{i}-{\hat{y}}_{i}\right)}^{2} \end{array}$$

where

(6) $$\begin{array}{c} \sigma^{2}=\frac{1}{n}\sum_{i=1}^{n}{\left(y_{i}-{\hat{y}}_{i}\right)}^{2}. \end{array}$$

The model with the smaller AIC value is considered to be better. The AIC value does not have an absolute meaning, but it penalizes models for greater complexity, favoring simpler models when both fit the data similarly.

## 3. Results

**Figure 2:**
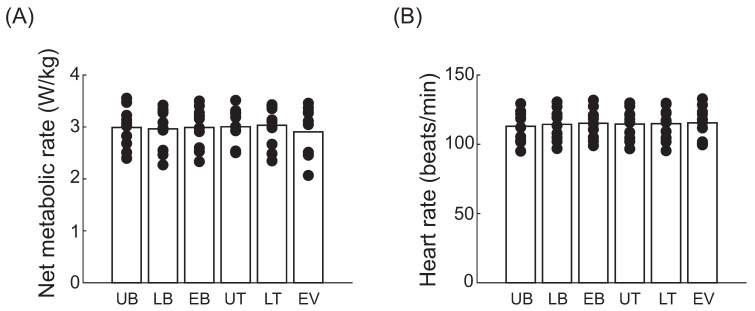
Net metabolic rate (**A**) and heart rate (**B**) of participants walking at 5 km/h with a 10 kg vest load under various load positions: upper back (UB), lower back (LB), entire back (EB), upper torso (UT), lower torso (LT), and entire vest (EV).

The mean, standard deviation and coefficient of variation values for metabolic rate and heart rate in each load position, each load weight, and each gait condition are shown in Table 1. There were no significant differences in the metabolic rate (*p* = 0.989) or heart rate (*p* = 0.998) across different load positions during walking with a 10 kg vest load (Figure 2).

**Figure 3:**
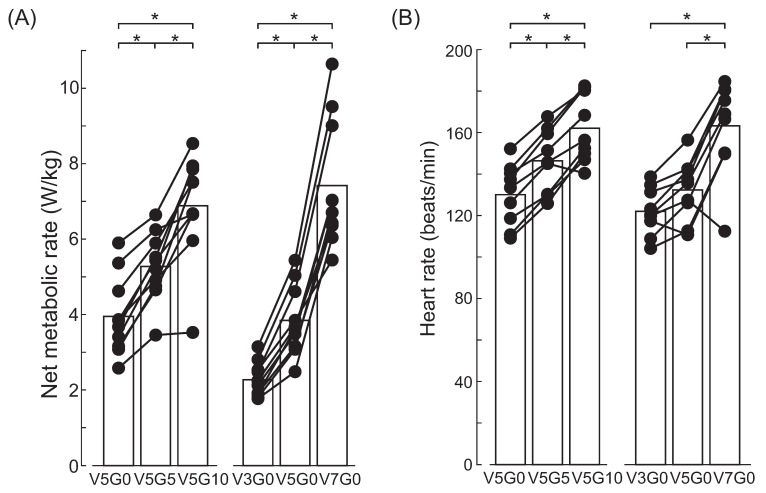
Net metabolic rate (**A**) and heart rate (**B**) of participants walking with a 10 kg vest load at various gait conditions. * indicates a significant difference between the two conditions.

The net metabolic rate increased significantly with the rise in walking incline and speed (*p* < 0.001, Figure 3). Significant differences in net metabolic rate were observed between any two inclines (or speeds) (*p* = 0.05). Similarly, heart rate increased significantly with the increase in walking incline and speed (*p* < 0.001). The heart rate at any given incline was significantly different from that at the other two inclines (*p* = 0.05). Additionally, the heart rate during level walking at 7 km/h was significantly higher than at 5 km/h and 3 km/h (*p* = 0.05).

**Figure 4:**
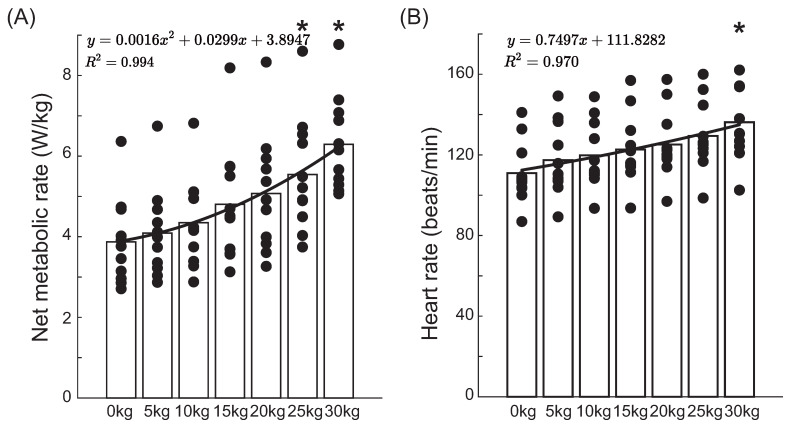
Net metabolic rate (**A**) and heart rate (**B**) of participants walking with various load mass conditions at 5 km/h. * indicates a significant difference between this condition and the 0 kg condition.

Both net metabolic rate and heart rate increased significantly with the increase in load (*p* < 0.001, Figure 4). The net metabolic rate at 25 kg and 30 kg was significantly higher compared to the unloaded walking condition (*p* = 0.05), and the heart rate at 30 kg was also significantly higher compared to the unloaded walking condition (*p* = 0.05).

The net metabolic rate exhibited a quadratic relationship with the vest load weight. The AIC of the quadratic model was lower than that of the linear model, and the $R^{2}$ of the quadratic model was closer to 1 than that of the linear model (Table 2). In contrast, heart rate displayed a linear relationship with the vest load weight. While the $R^{2}$ of the quadratic and linear models were identical, the AIC of the quadratic model was bigger than that of the linear model.

## 4. Discussion

This study explored the effects of load mass, load position, walking incline, and walking speed on metabolic rate and heart rate during weighted vest walking. The findings indicated that the load position of the vest had no significant impact on physiological responses. Metabolic rate increased quadratically with load mass, while heart rate increased linearly with load mass. Furthermore, both metabolic rate and heart rate significantly increased with walking incline and speed. These results provide insights for optimizing load carriage strategies in occupational and athletic training contexts.

In this study, no significant differences in metabolic rate or heart rate were found across various vest load positions. A plausible explanation for this finding is that the vest load was tightly adhered to the torso, minimizing major biomechanical changes that might otherwise influence metabolic cost. The vertical and anterior–posterior distributions of backpack load on the torso can influence metabolic cost. Lower load placements have been associated with increased forward lean, resulting in higher energy expenditure compared to higher placements [35,36]. Similarly, distributing the load between the front and back of the torso has been shown to reduce forward lean compared to back-only loading, although no significant differences in metabolic rate have been observed [5]. Previous studies have shown that backpack load position does not significantly affect heart rate [12,23]. Vest loading position in this study also had no significant effect on heart rate.

Walking speed can influenced the sensitivity of metabolic responses to different load positions. A study [25] reported that at walking speeds less than 3.6 km/h and greater than 4.8 km/h, there were no significant differences in metabolic cost between upper and lower body load positions. This is consistent with the findings of the current study, where no significant differences in metabolic rate were observed at a walking speed of 5 km/h for different load positions. Our results further corroborated these findings, showing no significant differences among the upper back, lower back, entire back, upper torso, lower torso, or entire vest load positions at this speed.

Heavier loads require more metabolic cost and impose greater cardiovascular demand. This is consistent with the majority of previous studies, which have shown that both metabolic rate and heart rate increase with load mass [22]. The quadratic relationship between load mass and metabolic cost, as indicated by the lower AIC values and higher $R^{2}$ of the quadratic model, suggests that incremental metabolic costs increase disproportionately with heavier loads. This finding differs from the linear relationship between absolute load mass and metabolic rate observed in some backpack load studies [8], as well as the linear relationship between relative load mass (normalized to body mass) and metabolic rate reported in other studies [37,38]. These discrepancies highlight the potential influence of load distribution, mechanical constraints, and experimental design on the metabolic cost–load relationship. The presence of a quadratic term in the vest load condition may be attributed to the mechanical constraints imposed on the thorax by the load, which could affect the cardiopulmonary system’ s ability to sustain prolonged activity. Tight-fitting vests or body armor, similar to the weighted vest used in this study, have been shown to mechanically restrict lung expansion, thereby limiting tidal volume and reducing maximum work capacity, which in turn increases oxygen consumption during submaximal tasks [39,40].

This finding differs from the linear relationship between absolute load mass and metabolic rate observed in some backpack load studies [5], as well as the linear relationship between relative load mass (normalized to body mass) and metabolic rate reported in other studies [37,38]. These discrepancies highlight the potential influence of load distribution, mechanical constraints, and experimental design on the metabolic cost–load relationship.

An observation in this study is the greater variability in metabolic rate compared to heart rate across different load positions, load masses, and gait conditions. The coefficient of variation (CV) for metabolic rate was consistently higher than that for heart rate, indicating that metabolic rate exhibits more inter-individual variability. The variability in metabolic rate was greatest when the load was uniformly distributed across the entire vest. This suggests that even with uniform load distribution, individual differences in biomechanics and physiology can lead to substantial differences in energy expenditure. As load mass increased, the standard deviation of metabolic rate tended to increase but not as rapidly as the load mass itself, resulting in a decreasing CV trend. This observation is consistent with [41], which reported that as load mass increased during backpack loading, variability in metabolic rate also increased.

The results of this study provide practical insights for optimizing load-carrying equipment in occupations requiring sustained physical activity under load. Our findings indicate that the placement of loads on the torso has a limited impact on metabolic costs, suggesting that weighted vests should prioritize even weight distribution to minimize strain on specific body regions. However, as load mass increases, metabolic costs rise disproportionately, underscoring the need to minimize total load to reduce physiological strain. Considering the significant effects of walking incline and speed on both metabolic and cardiovascular responses, load-bearing systems should account for terrain and movement speed in real-world applications. Lighter-load configurations or the use of assistive technologies, such as exoskeletons, may be particularly beneficial in high-intensity environments that require steep inclines or rapid movement.

In this study, while we have made efforts to control for the potential influence of anthropometric data on metabolic responses, we acknowledge that factors such as body mass, height, and body composition may indeed influence the results to some extent. As stated in Equation (2), all metabolic rates were normalized by individual body mass to account for baseline physiological differences. The narrow anthropometric range of participants was intentionally maintained to minimize confounding effects, as extreme body dimensions can disproportionately affect load-carriage energetics. This design choice enhances internal validity while acknowledging the need for future studies with broader demographics.

There are several limitations in this study. First, it was conducted exclusively on healthy young male participants, limiting the generalizability of the findings to other populations, such as women or older adults. Future research should include more diverse participant groups to better understand the differential effects of load carriage on various populations. Additionally, the experiments were conducted in a controlled laboratory environment, which may not fully replicate real-world conditions, such as varied terrain, weather, or prolonged fatigue. Future studies could explore the long-term effects of load carriage in more realistic field conditions to better inform equipment design. Finally, while this study explored different load positions and masses, it did not investigate the potential benefits of dynamic load-shifting techniques or other modern innovations in load-bearing equipment. Future research could explore how these techniques might mitigate the physiological costs observed in our trials. In conclusion, this study highlights the importance of considering load mass and distribution in designing load-bearing equipment. While load position on the torso has minimal impact on energy expenditure, total load mass plays a critical role in determining metabolic costs. These findings can inform the development of more effective and safer load-bearing strategies for occupational and athletic contexts.

## 5. Conclusions

This study investigated the effects of load mass, load placement, walking incline, and walking speed on metabolic rate and heart rate during walking with a vest load. The findings revealed that while the position of the load had no significant impact on either metabolic rate or heart rate at a moderate load of 10 kg, both physiological measures increased significantly with heavier loads. Notably, the metabolic rate exhibited a quadratic relationship with load mass, suggesting that the energy cost escalates non-linearly as the load increases. In contrast, heart rate showed a linear increase, indicating a proportional cardiovascular response to the load. Additionally, both metabolic rate and heart rate were significantly affected by walking conditions, with substantial increases observed at higher inclines and speeds. These results underscore the importance of considering both load mass and walking conditions when assessing the physiological demands of load carriage. Overall, this study enhances our understanding of the factors influencing metabolic and cardiovascular responses during walking with a load. The insights gained from this research can inform the design of training programs and occupational guidelines, optimizing safety and performance. Future studies should consider a broader range of populations and load conditions to further elucidate these complex physiological responses.

## Figures and Tables

**Table 1:** Mean, standard deviation (SD) and coefficient of variation (CV) values for metabolic rate (W/kg) and heart rate (beats/min) in each load position, each load weight, and each gait condition.

	Metabolic Rate			Heart Rate		
	Mean	SD	CV (%)	Mean	SD	CV (%)
Load position						
UB	2.99	0.37	12.37	112.95	10.69	9.46
LB	2.96	0.38	12.84	114.37	10.59	9.26
ULB	2.99	0.37	12.37	115.13	10.28	8.93
UBC	3.00	0.35	11.67	114.62	10.60	9.25
LBC	3.03	0.38	12.54	114.81	11.12	9.69
ULBC	2.90	0.46	15.86	115.40	11.20	9.71
Load weight						
0 kg	3.87	1.02	26.45	110.98	14.55	13.11
5 kg	4.09	1.05	25.66	117.42	17.02	14.50
10 kg	4.35	1.06	24.28	119.77	15.80	13.19
15 kg	4.80	1.38	28.74	122.64	16.63	13.56
20 kg	5.07	1.39	27.49	125.11	16.07	12.84
25 kg	5.54	1.34	24.25	129.39	16.40	12.67
30 kg	6.29	1.10	17.48	136.20	17.30	12.70
Gait condition						
V5G0	3.95	1.00	25.20	130.06	14.04	10.79
V5G5	5.27	0.86	16.30	146.35	14.35	9.81
V5G10	6.88	1.34	19.41	162.12	15.46	9.53
V3G0	2.27	0.44	19.31	122.04	10.81	8.86
V5G0	3.84	0.88	22.78	132.30	13.86	10.47
V7G0	7.42	1.61	21.74	163.26	21.57	13.22

**Table 2:** AIC and $R^{2}$ of the linear model and the quadratic model for net metabolic rate and heart rate.

	Net Metabolic Rate		Heart Rate	
	Linear	Quadratic	Linear	Quadratic
AIC	−2.49	−12.87	27.94	29.12
$R^{2}$	0.96	0.99	0.97	0.97

## Data Availability

The raw data supporting the conclusions of this article will be made available by the authors on request.
